# Modeling Stress-Recovery Status Through Heart Rate Changes Along a Cycling Grand Tour

**DOI:** 10.3389/fnins.2020.576308

**Published:** 2020-12-02

**Authors:** Anna Barrero, Anne Le Cunuder, Guy Carrault, François Carré, Frédéric Schnell, Solène Le Douairon Lahaye

**Affiliations:** ^1^University of Rennes 2, M2S Laboratory, Rennes, France; ^2^CHU Rennes, Inserm CIC 1414, Rennes, France; ^3^University of Lyon, Ecole Normale Supérieure, CNRS UMR 5672, Lyon, France; ^4^University of Rennes 1, Inserm, LTSI – UMR 1099, Rennes, France; ^5^University of Rennes, CHU Rennes, Inserm, LTSI – UMR 1099, Rennes, France

**Keywords:** females, cycling, endurance, mathematical model, performance, heart rate variability, stress-recovery status

## Abstract

**Background:**

Heart rate (HR) and HR variability (HRV) indices are established tools to detect abnormal recovery status in athletes. A low HR and vagally mediated HRV index change between supine and standing positions reflected a maladaptive training stress-recovery status.

**Objectives:**

Our study was focused on a female multistage cycling event. Its overall aim was twofold: (1) quantify the correlation between (a) the change in HR and HRV indices during an active orthostatic test and (b) subjective/objective fatigue, physical load, and training level indicators; and (2) formulate a model predicting the stress-recovery status as indexed by $\Delta \,\bar{R\,R}$ and ΔLnRMSSD (defined as the difference between standing and supine mean RR intervals and LnRMSSD, respectively), based on subjective/objective fatigue indicators, physical load, and training levels.

**Methods:**

Ten female cyclists traveled the route of the 2017 Tour de France, comprising 21 stages of 200 km on average. From 4 days before the beginning of the event itself, and until 1 day after its completion, every morning, each cyclist was subjected to HR and HRV measurements, first at rest in a supine position and then in a standing position. The correlation between HR and HRV indices and subjective/objective fatigue, physical load, and training level indicators was then computed. Finally, several multivariable linear models were tested to analyze the relationships between HR and HRV indices, fatigue, workload, and training level indicators.

**Results:**

HR changes appeared as a reliable indicator of stress-recovery status. Fatigue, training level, and $\Delta \,\bar{R\,R}$ displayed a linear relationship. Among a large number of linear models tested, the best one to predict stress-recovery status was the following: $\Delta \,\bar{R\,R}=$1,249.37+12.32V̇O_2__*max*_ + 0.36 km⋅week^–1^−8.83 HR_*max*_−5.8 RPE−28.41 perceived fatigue with an adjusted *R*^2^ = 0.322.

**Conclusion:**

The proposed model can help to directly assess the adaptation status of an athlete from RR measurements and thus to anticipate a decrease in performance due to fatigue, particularly during a multistage endurance event.

## Introduction

Physical training must combine workloads and recovery periods (Bishop et al., 2008). An optimal match between these two parameters is requested to avoid fatigue accumulation and thus reach the best individual physical performance level. Conversely, in the event of an imbalance between the two parameters, a state of overreaching or overtraining with marked drop in performance may occur (Kuipers, 1998). The individual optimal balance between work and recovery is difficult to achieve, especially for highly trained athletes. Thus, having relevant indicators of the exercise stress-recovery status could be a real advantage for individual athlete’s monitoring. The aim of this study was to provide a new tool for coaches to help them to assess the athlete’s individual fatigue level.

Several tools have been proposed to assess the fatigue level in daily routine of athletes (Buchheit, 2014; Halson, 2014). The most used are those providing an indirect evaluation of heart rate (HR) control by the autonomic nervous system (ANS) as analysis of spontaneous RR interval duration or HR variability (HRV) temporal and spectral indices (Bellenger et al., 2016), although the spectral ones present some limits (Buchheit, 2014). The HRV analysis can be performed either statically or dynamically, reflecting the heart adaptation to a physiological stress (Buchheit, 2014). Training-induced fatigue causes a more or less weak response of the ANS to external stimuli. Specifically, under normal training conditions (stress/recovery balance), intense strenuous exercise results in a decrease in vagally mediated HRV indices, followed by cardiac parasympathetic reactivation, which takes place during the recovery period (24–48 h) during which the cardiovascular system plays an important role in restoring body’s homeostasis (Stanley et al., 2013). In training with a stress/recovery imbalance, a more marked and prolonged alteration of only vagally mediated HRV indices is reported (Bosquet et al., 2008; Plews et al., 2012). These results seem to support a link between fatigue and mainly the parasympathetic nervous system. Thus, HRV analysis, and especially focusing on parasympathetic’ activity, represents a noninvasive method to track and record training status, “exercise readiness,” and post-exercise fatigue in athletes (Buchheit, 2014; Bellenger et al., 2016). Indeed, in the parasympathetic system presenting the more marked impact on the post-exercise recovery, the vagally mediated HRV index variation is able to evaluate the cardiac response (Michael et al., 2017): the root-mean-square difference of successive normal RR intervals (RMSSD or its natural log, LnRMSSD) in the time domain is the most recommended (Plews et al., 2012; Buchheit, 2014; Bellenger et al., 2016), since it represents pure parasympathetic HR modulation (Malik and Camm, 1993).

However, it seems that the correct interpretation of HR or HRV fluctuations during the training period requires the comparison of these markers with other objective signs of fatigue to detect the risk of overreaching or overtraining (Stanley et al., 2013; Buchheit, 2014). From nocturnal ANS activity recordings in swimmers, Chalencon et al. (2012) found strong relationships between resting high-frequency (HF) power of HRV and 400-m freestyle time-trial performance of the next morning (Chalencon et al., 2012). The higher the HF power values, the better the performance. Moreover, using the same protocol of ANS activity and performance recordings, the authors demonstrated that intensive training periods have a negative impact on both performance and HF due to fatigue (negative influence). In addition, modeling the effect of a 30-week training on swimmers’ performance allowed for an accurate prediction of individual performance (Chalencon et al., 2015), supporting the relevance of a mathematical modeling of HRV in order to predict responses to training. To our knowledge, such mathematical analysis was only conducted over long training periods. Therefore, it appears relevant to also assess daily athlete’s response on a shorter timescale, such as during a competitive event spreading over a few weeks (e.g., Grand Tours in cycling and World Football Cup) or during training camps, in which stress and fatigue affect day-to-day performances.

In a previous study on well-trained female cyclists, we have demonstrated that during a multistage event, HR and HRV indices evolved along the event in correlation with the daily physical load (Barrero et al., 2019). Briefly, we have observed a progressive increase of resting HR during the event and a progressive imbalance in the autonomic balance with an increase in the low-frequency (LF) power value that partly reflects the effects of sympathetic tone and a decrease in the HF and RMSSD values that reflects the parasympathetic effects. Our results also highlighted that variation in HR and HRV indices when changing from supine-to-standing position during an active orthostatic test is strongly correlated with the fatigue status (Barrero et al., 2019). The use of active orthostatic test has been recommended to detect fatigue in athletes, because of its ability to detect autonomic responses not observed with isolated supine or standing measures (Bosquet et al., 2008; Schmitt et al., 2015). This test explores the reactivities of sympathetic (excitation) and parasympathetic systems (withdrawal) in response to the position change (Taylor, 1994) and is the most widely used physiological maneuver for assessing neuro-vegetative and cardiovascular responsiveness. When standing from a supine position, the normal response is an increase in HR to maintain blood pressure (Tse et al., 2005). In well-trained athletes, with respect to supine rest values, after active stand-up, a marked increase of HR associated with a decrease in global HRV, HF, and RMSSD and with an increase of LF/HF ratio has been reported (Hynynen et al., 2008). These observations suggest that standing up normally induced mainly parasympathetic withdrawal. In overtrained athletes, an attenuation of parasympathetic and sympathetic activity during both supine and standing positions has been observed. Moreover, in these athletes, in response to stand-up position, the HR increases, and the decrease of total power, HF, LF, and RMSSD seemed lower than in non-overtrained athletes (Hynynen et al., 2008). Although a complex phenomenon (Schmitt et al., 2015), it is proposed that training induced-fatigue attenuates baroreflex response to change in position (Uusitalo et al., 2000). Thus, a low HR change between supine and standing positions reflects a maladaptive training stress-recovery status.

The multidimensional monitoring of recovery status has been underlined (Heidari et al., 2018), and it was recommended to associate other markers with HR/HRV indices to more accurately detect the state of overtraining (Stanley et al., 2013; Buchheit, 2014). Because progression to overtraining syndrome appears to be associated with psychological states, the use of self-administered questionnaires on perceived physical and psychological well-being (WB) by athletes has been recommended (Saw et al., 2016). In this context, monitoring the evolution of several indices such as rate of perceived exertion (RPE), the WB, the sleep quality, and the delayed-onset muscle soreness (DOMS) during intensive periods of training has been proposed (Ouergui et al., 2020).

In our previous study, we observed a progressive decrease of HR and HRV responses induced by the active orthostatic test. These alterations were positively associated with the athlete’s daily physical load and thus with fatigue (Barrero et al., 2019). However, the descriptive method we used to analyze the day-to-day HR and HRV indices did not allow to accurately predict the cyclist’s individual adaptation ability in response to the exercise performed.

Thus, based on these previous data, the aim of the present study was first to investigate the individual correlation between the supine-standing difference of HR and HRV index values presented above, fatigue, physical load, and training level and second to formulate models relating two dependent HR variables ($\Delta \,\bar{R\,R}$ and ΔLnRMSSD defined as the difference between standing and supine mean RR intervals and LnRMSSD, respectively) to subjective/objective fatigue, physical load, and training level indicators in order to investigate the parameters better predicting $\Delta \,\bar{R\,R}$ and ΔLnRMSSD. The added value of the current study is to propose a model allowing coaches to understand the adaptation ability of athletes to a multistage endurance event and helping them to anticipate a decrease in performance due to fatigue.

The design of our study has been based on two assumptions.

First, a relation exists between ANS alterations, mainly the cardiac vagal tone and early fatigue detection in athletes. Indeed, the development of fatigue in athletes is considered as a continuum process with, one side, the voluntary and controlled fatigue and, the opposite side, the uncontrolled fatigue, so-called overtraining (Meeusen et al., 2013). All the states of fatigue in athletes classically associate the same more or less marked symptoms: decrease in physical performance, neuro-endocrinal abnormalities, and psychological trouble such as irritability. Thus, the early detection of fatigue in athletes by use of HR and HRV analyses could be in line with the biological behavioral model (Grossman and Taylor, 2007). In accordance with previously published data concerning this first hypothesis, we assumed that due to the repetition of the successive endurance stages, we would observe a lower change of HR and of HR vagally mediated HRV between supine and standing positions during the orthostatic test. Moreover, we also hypothesized that HR and time-domain HRV vagal indices would present the higher relation with fatigue parameter levels.

Second, based on previous research (Ouergui et al., 2020), we hypothesized that the most suitable prediction models for both $\Delta \,\bar{R\,R}$ and ΔLnRMSSD should include parameters related on the one hand with physical performance, such as amount of training and level of performance, and on the other hand with levels of WB and of exertion feeling. We hypothesized that parameters of V̇O_2__*max*_, WB, RPE, sleep quality, and DOMS could be included in the prediction model.

## Materials and Methods

The first results of this prospective study have been previously published (Barrero et al., 2019).

This scientific project took place as part of the sports project “Donnons des elles au vélo J-1” aimed to promote women’s cycling. This sport project included only 11 well-trained female cyclists, and of them, 10 participated in the whole study.

### Population

All were healthy, with no medical history of cardiovascular disease, and were not currently taking medication. At the time of this study, their weekly training mileage ranged from 100 to 250 km per week. All cyclists had a minimum of 2 years of competitive cycling experience. The characteristics of the athletes already presented (Barrero et al., 2019) are recalled in Table 1.

**TABLE 1:** Anthropometric and physical performance characteristics of the cyclists (mean ± SD).

Characteristic	Value
Age (years)	31.7 ± 4.7
Weight (kg)	57.0 ± 6.3
Height (m)	1.60 ± 0.1
BMI (kg⋅m^–2^)	21.4 ± 1.9
V̇O_2__*max*_ (ml⋅min⋅kg^–1^)	53.6 ± 5.2
Maximal power output (W)	285.5 ± 19.2
Relative maximal power output (W kg^–1^)	5.0 ± 0.5
Maximal HR (bpm)	185.7 ± 7.2
Previous cycling experience (years)	14.0 ± 8.9
Current training level (km⋅week^–1^)	187.5 ± 51.5

*BMI, body mass index; V̇O_2__*max*_, maximal oxygen uptake; HR, heart rate.*

This study received the approval of our hospital ethics committee. After information was given, all participants provided written informed consent. The study was conducted in accordance with the “Good Clinical Practice” guidelines as laid down in the Declaration of Helsinki.

### Cycling Event

The characteristics of the multistage cycling event were previously described (Barrero et al., 2019). In brief, cyclists performed the 21 stages of men’s 2017 Tour de France (TdF) 1 day before each stage of the official race. All event stages were performed without any spirit of competition or performance goal. The unique cyclists’ objective was to complete 3,540 km of the TdF to promote women’s cycling.

### Preliminary Testing

Each cyclist had a preparticipation medical evaluation with a clinical exam, a resting ECG (Mac 1600, GE Healthcare, Chicago, IL, United States) and an incremental maximal cardiopulmonary exercise test performed on an electronically braked cycle ergometer (Excalibur Sport, Lode, Netherlands) with continuous ECG and blood pressure monitoring and gas exchange analysis (Case system-Power cube, GE Healthcare, Chicago, IL, United States). The French Cycling Federation incremental exercise protocol was used. It started with a warm-up period (100 W for 5 min and 150 W for 1 min) followed by a step load-increase of 25 W min^–1^ until exhaustion. This preliminary testing took place 1 week before the first stage.

### RR Interval Recording and Analysis

The RR interval recording and HRV analysis protocols used were previously described (Barrero et al., 2019). Baseline pre-TdF RR intervals were collected daily during 4 days before the event to obtain a basal HRV state. Then RR recordings were performed every day of the multistage event. All resting recordings were made in the morning fasting, right after awakening, before the cyclist gets up. In order to avoid mental activity and stress and thus to place the cyclists in an optimal physiological rest state, RR recordings were performed in spontaneous breathing (Bernardi et al., 2000).

Briefly, all RR interval samples were recorded with a portable HR monitor (Polar V800, Kempele, Finland) during the two successive phases of the test: 7 min in a supine position followed by 7 min in standing position as recommended (Bourdillon et al., 2017). Individual RR recorded data were downloaded via Polar FlowSync software for mac version 2.6.4 (Polar, Kempele, Finland) and exported for later analysis. The Kubios HRV Standard software version 3.0.0 2 (Biosignal Analysis and Medical Imaging Group at the Department of Applied Physics, University of Kuopio, Kuopio, Finland) was used. For the HRV analysis, the last 5-min window for each position was used. All the ectopic beats were filtered with the artifact correction option of the software. A very low threshold was applied when needed (<5% of corrected beats). Both time and frequency domain HRV analysis were performed. The HRV spectrum is calculated with fast Fourier transform-based Welch’s periodogram for spectral analysis. The RMSSD, which reflects cardiac vagal tone, was calculated. The HF (0.15–0.40 Hz) and LF (0.04–0.15 Hz) domains were analyzed. The HF band reflects cardiac vagal tone, while the LF band indicates both sympathetic and parasympathetic influences. RMSSD, HFnu, LFnu (normal units) absolute values, and their difference between supine and standing positions were calculated. The difference of the natural logarithm LnRMSSD between supine and standing positions was also studied. The normalized (or normalized unit) spectral indices are defined by the developers of the Kubios HRV Standard software v3.0.0 2 as HFnu = HF/(LF + HF) and LFnu = LF/(LF + HF) (Biosignal Analysis and Medical Imaging Group at the Department of Applied Physics, Kuopio, Finland) in accordance with the recommendations (No authors listed, 1996).

### Daily Collection and Analysis of Heart Rate and Workload

#### Heart Rate and GPS Recording

Both HR and GPS data were continuously registered with the Polar V800 (Polar, Kempele, Finland) portable monitor during each stage.

#### Workload Evaluation

The objective load of the daily individual exercise performed was estimated from the HR collected during each stage. For this, the recorded HR values were divided into five zones according to the percentages (i.e., 50–60, 60–70, 70–80, 80–90, and 90–100%) of the individual maximum HR obtained during the maximum effort test. Then, the individual daily internal workload was estimated using the training pulse score (TRIMP) method (Edwards, 1993). In this model proposed by Edwards, the quantification of the workload is derived from the duration of the exercise maintained in the five HR zones described above (Edwards, 1993).

The individual RPE of the 21 stages was evaluated with Borg CR-10 scale within 30 min after each stage (Borg, 1990). RPE was indicative of the subjective load of each stage.

### Perceived Fatigue Evaluation (Well-being Questionnaire)

Cyclists answered every morning, during breakfast, a questionnaire with four questions focusing on perceived general fatigue, sleep quality, DOMS, and stress level. Each question scored on a 7-point (with 1 and 7 representing poor and very good WB ratings, respectively) scale. Overall WB was determined by summing the four questions’ scores (Hooper et al., 1995).

### Statistical Analysis

The first step of the statistical analysis concerned the first hypothesis of the study. We studied the evolution of HR and HRV indices throughout the cycling event in our previously published study (Barrero et al., 2019). Then, we investigated the correlation between individual HR (RR interval duration) and HRV indices, and subjective/objective fatigue, physical load, and training level indicators, in order to establish the best fatigue marker. We analyzed more precisely the RMSSD of RR interval duration in supine (RMSSDsup) and in standing (RMSSDsta) positions, the difference ΔRMSSD = RMSSDsup−RMSSDsta and its natural logarithm (ΔLnRMSSD), the LF and HF indices of RR time series, and the ratio LF/HF. We also computed the RR duration mean values in supine (MeanRRsup) and standing (MeanRRsta) positions. Finally, the difference of the mean time interval between two successive heart beats ($\Delta \,\bar{R\,R}$) between supine and standing positions was computed (White, 1980; Tse et al., 2005): $\Delta \,\bar{R\,R}=$RRsupine−RRstanding.

We then calculated the correlation coefficient between each of these indices and subjective/objective fatigue, physical load, and training level indicators (see section “Materials and Methods”).

The second step of the analysis concerned the second hypothesis of the study. To test this second hypothesis, we first had to characterize the influence of the cyclist’s training level on her adaptation all along the multistage event. To do this, the impact of each cycling stage was evaluated through the daily change observed on WB, with ΔWB = WB_(stage t)_−WB_(stage t–1)_. A low daily ΔWB indicates that the cyclist was slightly impacted by the stage. Then, all daily ΔWB was averaged and plotted for each cyclist as a function of her weekly pre-TdF training load (km⋅week^–1^).

The final step of the statistical analysis concerned the second hypothesis of the study. We aimed to test models to investigate which objective/subjective fatigue, physical load, and training level indicators best predict $\Delta \,\bar{R\,R}$ and ΔLnRMSSD. For this purpose, different multivariable linear models (LMMs) were tested in order to estimate which set of variables best explains the $\Delta \,\bar{R\,R}$ and ΔLnRMSSD.

For both, each model was a linear combination of up to eight parameters related to training and physical condition (km⋅week^–1^, v̇O_2__*max*_, HR_*max*_) on the one hand and to the perceived difficulty of the cycling stage and its impact on the cyclist (RPE, DOMS, perceived fatigue, quality of sleep, and stress) on the other hand:

$\Delta \,\bar{R\,R}$ = *f* (km⋅week^–1^, V̇O_2__*max*_, HR_*max*_, RPE, perceived fatigue, DOMS, quality of sleep, stress)

where *f* is a linear function.

ΔLnRMSSD = *f* (km⋅week^–1^, V̇O_2__*max*_, HR_*max*_, RPE, perceived fatigue, DOMS, quality of sleep, stress)

where *f* is a linear function.

The training level, assessed trough V̇O_2__*max*_ and km⋅week^–1^, clearly influences the fatigue accumulated during the event.

For each parameter, we thus tested all 2^8^ = 256 possible models, covering all possible combinations of the eight parameters. The predictive power of each model was estimated through its Akaike information criterion (AIC) and the highest adjusted R^2^, which penalize for including extra fitting parameters (Akaike, 1974). The best model corresponds to the lowest AIC value. In particular, if two models have the same R^2^, the one having the less parameters has the lowest AIC.

The statistical significance of each parameter was estimated through its *p*-value with a significant value stated at *p* < 0.05. The dispersion of our data did not allow us to evaluate nonlinear effects beyond LMMs.

All statistical analyses were performed with Python 3.7 software version.

## Results

All participants cycled for 21 consecutive stages, including two resting days. They all successfully completed the whole circuit of the TdF 2017 (3,540 km) (Barrero et al., 2019).

### Effects of Cycling Event on Heart Rate and Heart Rate Variability Indices

**FIGURE 1:**
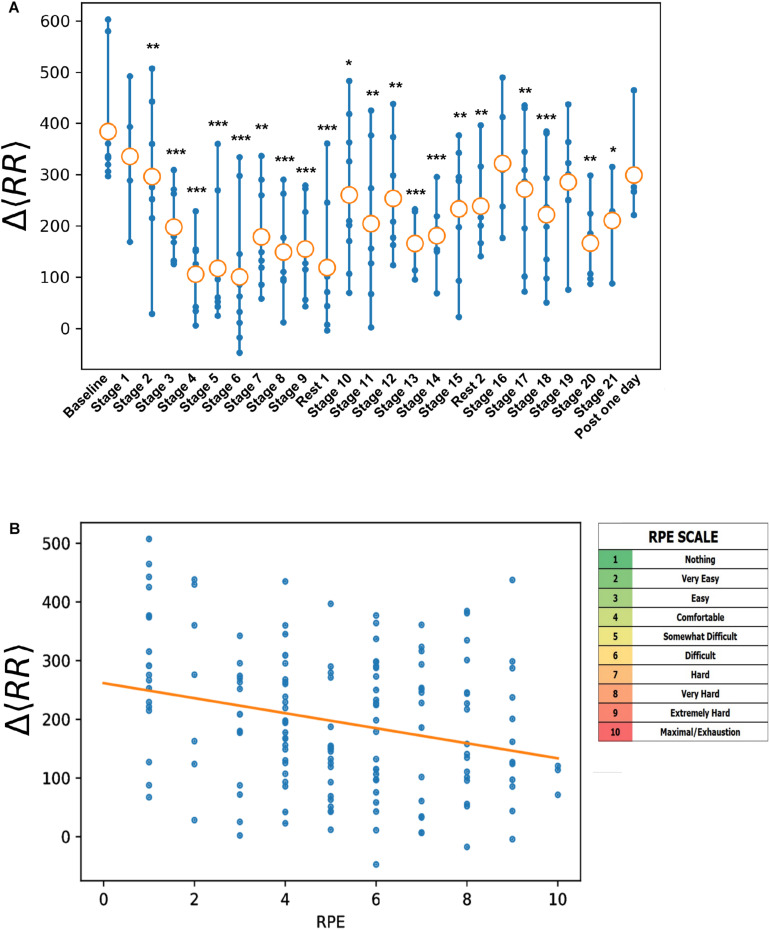
Evolution of $\Delta \,\bar{R\,R}$ stage by stage **(A)** and with daily workload assessed through rate of perceived exertion (RPE) score on CR-10 Borg scale **(B)**. **(A)** $\Delta \,\bar{R\,R}$ of stage *n* reflect stress/fatigue induced by stage n–1. Statistical differences with baseline: *p < 0.05, **p < 0.01, and ***p < 0.001.

The evolution of HR and HRV indices throughout the cycling event has been previously published (Barrero et al., 2019). To summarize, resting supine HR increased progressively in comparison with its basal value during the multistage event, and the HR value returned to its basal values after each rest day. On the other hand, standing HR values showed no significant evolution during the cycling event. All along the multistage event, we observed a progressive decrease in $\Delta \,\bar{R\,R}$. Indeed, compared with its initial value (day 0), a progressive decrease in $\Delta \,\bar{R\,R}$ was observed through successive stages. A small increase in $\Delta \,\bar{R\,R}$ was noted after each resting day (days 10, 17, and 24) (Figure 1A).

Regarding HRV, a progressive imbalance in the autonomic balance marked with a decrease in cardiac vagal activity, evaluated through RMSSD and HF, was noted all along the cycling event. The daily RMSSD standing–supine difference was lower than the basal value during the multistage event.

### Correlations Between Heart Rate and Heart Rate Variability Indices, Fatigue, Physical Load, and Training Levels Indicators

These correlations concern our first hypothesis. Table 2 shows correlations between HRV and subjective/objective fatigue, physical load, and training level indicators. As expected, MeanRRsup, MeanRRsta, ΔRMSSD, ΔLnRMSSD, and $\Delta \,\bar{R\,R}$ were negatively correlated with workload. We also observed significant negative correlations between RMSSDsup and workload indicators.

**TABLE 2:** Correlation coefficients (and their *p*-values) between heart rate (RR) and heart rate variability indices and subjective/objective fatigue, physical load, and training levels indicators.

	MeanRRsup	MeanRRsta	RMSSDsup	RMSSDsta	<ΔRR>	ΔRMSSD	ΔLn RMSSD	LF(nu)	HF(nu)	LF(ms^2^)	HF(ms^2^)	LF/HF
KMS	−0.44 (*p* = 0.04)	−0.34 (*p* = 0.04)	−0.35 (*p* = 0.02)	0.21 (*p* = 0.35)	−0.41 (*p* = 0.07)	−0.43 (*p* = 0.05)	−0.41 (*p* = 0.06)	−0.07 (*p* = 0.77)	0.07 (*p* = 0.77)	0.15 (*p* = 0.51)	0.13 (*p* = 0.58)	0.09 (*p* = 0.70)
TRIMPS	−0.27 (*p* = 0.001)	−0.12 (*p* = 0.175)	−0.20 (*p* = 0.019)	−0.05 (*p* = 0.532)	−0.22 (*p* = 0.009)	−0.20 (*p* = 0.020)	−0.23 (*p* = 0.005)	0.07 (*p* = 0.415)	−0.07 (*p* = 0.408)	−0.13 (*p* = 0.112)	−0.14 (*p* = 0.100)	0.06 (*p* = 0.465)
RPE	−0.17 (*p* = 0.033)	−0.04 (*p* = 0.574)	−0.26 (*p* = 0.001)	−0.06 (*p* = 0.444)	−0.26 (*p* = 0.001)	−0.17 (*p* = 0.029)	−0.16 (*p* = 0.042)	−0.10 (*p* = 0.200)	−0.11 (*p* = 0.150)	0.05 (*p* = 0.545)	−0.05 (*p* = 0.538)	−0.01 (*p* = 0.900)
Perceived fatigue	0.10 (*p* = 0.184)	0.20 (*p* = 0.011)	−0.17 (*p* = 0.023)	0.21 (*p* = 0.006)	−0.39 (*p* < 0.001)	−0.01 (*p* = 0.903)	−0.24 (*p* = 0.002)	0.11 (*p* = 0.169)	0.13 (*p* = 0.091)	−0.02 (*p* = 0.756)	0.02 (*p* = 0.759)	0.03 (*p* = 0.701)
DOMS	0.16 (*p* = 0.032)	0.23 (*p* = 0.003)	−0.26 (*p* = 0.001)	0.10 (*p* = 0.195)	−0.40 (*p* < 0.001)	0.05 (*p* = 0.500)	−0.22 (*p* = 0.004)	0.21 (*p* = 0.005)	0.20 (*p* = 0.009)	−0.01 (*p* = 0.906)	0.01 (*p* = 0.904)	−0.02 (*p* = 0.797)
Quality of sleep	0.24 (*p* = 0.002)	0.21 (*p* = 0.007)	−0.16 (*p* = 0.042)	−0.09 (*p* = 0.259)	−0.13 (*p* = 0.107)	0.14 (*p* = 0.080)	−0.06 (*p* = 0.447)	0.29 (*p* = 0.000)	0.22 (*p* = 0.005)	−0.00 (*p* = 0.988)	0.00 (*p* = 0.993)	−0.09 (*p* = 0.248)
Stress	0.15 (*p* = 0.056)	−0.14 (*p* = 0.069)	−0.41 (*p* = 0.000)	−0.28 (*p* = 0.000)	−0.24 (*p* = 0.001)	−0.07 (*p* = 0.392)	0.03 (*p* = 0.696)	−0.14 (*p* = 0.063)	−0.13 (*p* = 0.097)	−0.01 (*p* = 0.872)	0.01 (*p* = 0.879)	−0.11 (*p* = 0.154)

*KMS, km of the daily stage; TRIMPS, training impulse; RPE, rate of perceived exertion; DOMS, delayed-onset muscle soreness.*

No correlation was observed between RMSSDsta and RPE (*r* = 0.19, *p* = 0.41), RMSSDsta and TRIMPS (*r* = 0.047, *p* = 0.84), nor RMSSDsta and distance of the stages (*r* = 0.21, *p* = 0.35). Lastly, the LF, HF, and LF/HF indices were not correlated with workload indicators (*r* < 0.15, *p* > 0.46). The ΔRMSSD index showed significant negative correlation with TRIMPS (*r* = −0.20, *p* = 0.02), RPE (*r* = −0.17, *p* = 0.03), and KMS (*r* = −0.43, *p* = 0.05). We also computed the correlations of ΔLnRMSSD with TRIMPS (*r* = −0.23, *p* = 0.009), RPE (*r* = −0.04, *p* = 0.12), KMS (*r* = −0.41, *p* = 0.06), perceived fatigue (*r* = −0.24, *p* = 0.002), DOMS (*r* = −0.22, *p* = 0.004), quality of sleep (*r* = −0.06, *p* = 0.447), and stress (*r* = −0.22, *p* = 0.004).

The $\Delta \,\bar{R\,R}$ index showed significant correlation with workload markers with negative correlations with TRIMPS (*r* = −0.22, *p* = 0.009), RPE (*r* = −0.26, *p* < 0.05), daily stage distance (*r* = −0.41, *p* = 0.07), perceived fatigue (*r* = −0.39, *p* < 0.001), DOMS (*r* = −0.40, *p* < 0.001), quality of sleep (*r* = −0.13, *p* = 0.107), and stress (*r* = −0.24, *p* = 0.001) (Table 2). Otherwise, the overall $\Delta \,\bar{R\,R}$ decrease observed all along the multistage cycling event was correlated (*r* = 0.41, *p* < 0.05) with the increase of RPE (Figure 1B).

Correlations between $\Delta \,\bar{R\,R}$, ΔRMSSD or ΔLnRMSSD, and subjective/objective fatigue, physical load, and training level indicators are statically comparable (the *p*-value of the William’s test we used to compare correlations is >0.1 for each indicator).

### Impact of Training Level

**FIGURE 2:**
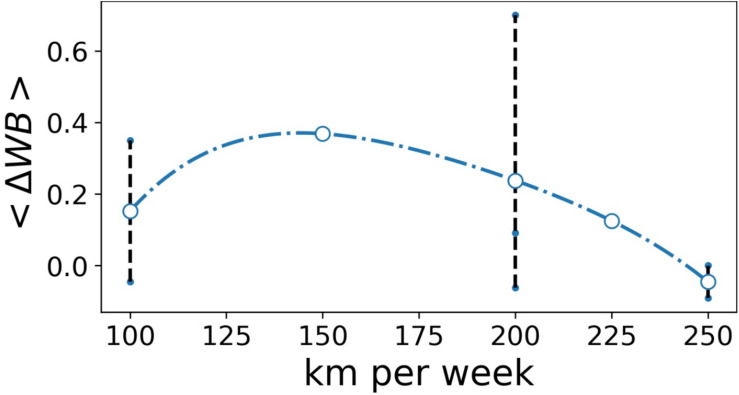
Averaged change in well-being (<ΔWB>) as a function of weekly training load (km week^– 1^). Blue circles represent change in well-being averaged on cyclists covering the same distance per week. The small blue points and the dotted lines represent individual change in WB.

To test the second hypothesis of the study, we first had to characterize the influence of the cyclist’s training level on her adaptation all along the multistage event, and we considered the change in mean WB on all the stages as a function of individual weekly training load. The results are shown in Figure 2. Overall, consequent to the accumulated fatigue, the feeling of WB gradually decreased all along the event. This decrease was less marked in the most trained cyclists. However, the relationship observed was not linear but curvilinear. Finally, we must emphasize the great individual variability of the evolution of WB for the same weekly training load (i.e., 100 or 200 km⋅week^–1^ in Figure 2).

### Multivariable Linear Models

To test our second hypothesis, we had lastly to consider 256 models for both $\Delta \,\bar{R\,R}$ and ΔLnRMSSD. Among them, the four presenting the lowest AIC and the highest adjusted R^2^ for $\Delta \,\bar{R\,R}$ and ΔLnRMSSD are shown in Tables 3, 4, respectively. The fitting coefficients are reported with their *p*-value.

**TABLE 3:** Linear coefficients, their corresponding *p*-values (in parentheses), adjusted *R*^2^ coefficients, and Akaike information criterion (AIC) of the different linear multivariable mixed models tested to explain the difference between standing and supine RR duration of cyclists.

	R1	R2	R3	R4	R5	R6	R7
V̇O_2__*max*_	12.32** (<0.001)	11.39** (<0.001)	12.59** (<0.001)	12.40** (<0.001)		11.78** (<0.001)	
Training load (km⋅week^–1^)	0.36 (0.021)	0.36 (0.019)	0.35 (0.023)	0.36 (0.023)	0.35 (0.033)		
HR_*max*_	−8.83** (<0.001)	−8.13** (<0.001)	−9.02** (<0.001)	−8.79** (<0.001)		−8.97** (<0.001)	−2.52 (0.075)
RPE	−5.90 (0.096)	−5.12 (0.16)	−5.99 (0.093)	−5.78 (0.156)	−4.71 (0.222)	−6.02 (0.094)	−5.94 (0.129)
Perceived fatigue	−28.41** (<0.001)	−24.55* (0.006)	−28.80** (<0.001)	−28.88 (<0.001)	−24.58* (0.009)	−29.62** (<0.001)	−19.32 (0.058)
DOMS		−6.20 (0.365)			−19.13* (0.004)		−19.33* (0.005)
Sleep quality				1.15 (0.85)			−10.81 (*p* = 0.10)
Stress			2.23 (0.748)				
Constant	1,249.37** (<0.001)	1,166.96** (<0.001)	1,266.30** (<0.001)	1,234.21** (<0.001)	314.60** (<0.001)	1,375.93** (<0.001)	860.54** (<0.001)
Adjusted R^2^	0.322	0.321	0.318	0.319	0.234	0.302	0.223
AIC	1,805	1,807	1,807	1,807	1,823	1,824	1,825

Adjusted *R*^2^ and AIC are used to estimate the predictive power of each model. These criteria are used instead of *R*^2^ in order to penalize including extra fitting parameters. Training load corresponds to the weekly pre-TdF training load of the cyclists. V̇O_2__*max*_, maximal oxygen uptake; HR_*max*_, maximal heart rate; RPE, rate of perceived exertion; WB, well-being; DOMS, delayed-onset muscle soreness. *p-value < 10^–2^, **p-value < 10^–3^.

**TABLE 4:** Linear coefficients, their corresponding *p*-values (in parentheses), adjusted *R*^2^ coefficients, and Akaike information criterion (AIC) of the different linear multivariable mixed models tested to explain the difference between standing and supine LnRMSSD of cyclists.

	R1″	R2″	R3″	R4″	R5″	R6″	R7″
V̇O_2__*max*_	0.042* (0.002)	0.026* (0.004)	0.024* (0.008)	0.038* (0.009)		0.040* (0.003)	
Training load (km⋅week^–1^)	0.002 (0.010)	0.002 (0.013)	0.002 (0.011)	0.002 (0.014)	0.0017 (0.036)		
HR_*max*_	−0.017 (0.094)			−0.014 (0.217)		−0.018 (0.081)	
RPE	−0.019 (0.308)	−0.019 (0.303)	−0.013 (0.487)	−0.015 (0.421)	−0.016 (0.407)	−0.020 (0.297)	−0.017 (0.392)
Perceived fatigue	−0.100 (0.013)	−0.114* (0.005)	−0.081 (0.083)	−0.084 (0.072)	−0.075 (0.118)	−0.108* (0.009)	0.085 (0.078)
DOMS			−0.044 (0.018)	−0.026 (0.464)	−0.059 (0.079)		−0.054 (0.109)
Sleep quality							
Stress	0.092 (0.012)	0.074 (0.034)	0.074 (0.033)	0.089 (0.016)	0.059 (0.092)	0.095 (0.011)	0.063 (0.078)
Constant	1.647 (0.238)	−0.495 (0.388)	−0.427 (0.457)	1.271 (0.393)	0.982** (<0.001)	2.365 (0.090)	1.331** (<0.001)
Adjusted *R*^2^	0.141	0.130	0.135	0.139	0.098	0.111	0.076
AIC	229.9	230.9	231.0	231.4	236.4	234.1	239.1

Adjusted *R*^2^ and AIC are used to estimate the predictive power of each model. These criteria are used instead of *R*^2^ in order to penalize including extra fitting parameters. Training load corresponds to the weekly pre-TdF training load of the cyclists. V̇O_2__*max*_, maximal oxygen uptake; HR_*max*_, maximal heart rate; RPE, rate of perceived exertion; WB, well-being; DOMS, delayed-onset muscle soreness. *p-value < 10^–2^, **p-value < 10^–3^.

It must be underlined that the V̇O_2__*max*_ value and the pre-TdF training load (km⋅week^–1^) that were included in all models appeared to be the most relevant parameters to explain the $\Delta \,\bar{R\,R}$ and the ΔLnRMSSD observed. To further quantify the importance of these indicators of the physical condition, we also included in Tables 3, 4 the best model, which does not include the V̇O_2__*max*_ (model 5), the number of km⋅week^–1^ (model 6), or none of them (model 7). Clearly, beyond the multistage event-induced fatigue, the physical condition is the key to account the $\Delta \,\bar{R\,R}$ and ΔLnRMSSD variations.

Specifically, the most relevant model to explain $\Delta \,\bar{R\,R}$ can be expressed as a multi-linear function:

$\Delta \,\bar{R\,R}$ = 1,249.37 + 12.32 V̇O_2__*max*_ + 0.36 km⋅week^–1^−8.83 HR_*max*_−5.8 RPE−28.41 perceived fatigue

where the *p*-values of each coefficient are given in Table 3.

And the best model to predict ΔLnRMSSD is:

ΔLnRMSSD = 1.647 + 0.042 V̇O_2__*max*_ + 0.002 km⋅week^–1^−0.017 HR_*max*_−0.019 RPE−0.100 perceived fatigue + 0.092 stress

where the *p*-values of each coefficient are given in Table 4.

Finally, to formulate a more accessible model for coaches and team managers, we looked for a linear relationship between $\Delta \,\bar{R\,R}$, a single indicator of fatigue and physiologic constants excluding the V̇O_2__*max*_, which is not easily measurable. This model can be expressed as $\Delta \,\bar{R\,R}=-$14.36 WB−2.06 HR_*max*_ + 0.35 km⋅week^–1^ + 689.62. The R^2^ adjusted and AIC observed for this model were, respectively, 0.194 and 2033.

Concerning ΔLnRMSSD, as we can see from adjusted R^2^, models predict less variance than for $\Delta \,\bar{R\,R}$ index (Table 4).

## Discussion

Intense physical training exposes the athlete to the risk of overreaching or of overtraining, partly due to an imbalance between training and recovery (Bishop et al., 2008). The information from overreaching and overtraining markers, especially based on HR and HRV analyses, seems reinforced when it is associated with other fatigue parameters (Stanley et al., 2013; Buchheit, 2014). Our study aimed at modeling the relationship between the level of fatigue reported by well-trained female cyclists during a multistage cycling event, their physical load, their training level, and the variations in HRV and HR rate indices in response to an active orthostatic test.

Regarding our first hypothesis, we noted a lower change of HR and of HR vagally mediated HRV between supine and standing positions during the orthostatic test. However, only HR and RMSSD, a time domain HRV index, were correlated with subjective/objective fatigue, physical load, and training level indicators. Lastly, the index $\Delta \,\bar{R\,R}$, defined as the difference between the average RR intervals measured in a supine position and then in a standing position, appeared a new indicator of stress/recovery status. These results confirmed our first hypothesis.

Regarding our second hypothesis, we have then demonstrated that $\Delta \,\bar{R\,R}$ and ΔLnRMSSD could be modeled as a linear function of training volume, V̇O_2__*max*_ and fatigue level, assessed through the RPE and the WB questionnaire. Thus, the results observed confirmed only partly our second hypothesis, because DOMS and sleep quality previously proposed (Ouergui et al., 2020) did not provide major information to specify the stress-recovery status in the multistage of endurance event studied (Table 3). Regarding ΔLnRMSSD, models predict less variance than for $\Delta \,\bar{R\,R}$ index. This result underlines the interest of $\Delta \,\bar{R\,R}$ in stress-recovery status prediction.

### Respective Values of Heart Rate Variability Indices

From all the HRV indices we used, time domain’s is the one that was the best correlated with workload and fatigue parameters (Table 2). Limits of spectral indices have been previously reported (Buchheit, 2014). Our results confirm also that the time-domain markers of parasympathetic effects seemed to be better adapted to explore fatigue level (Buchheit, 2014; Fazackerley et al., 2019). This can be explained by the fact that parasympathetic nervous system is implied in self-regulation mechanisms, which are critical for adaptation (Laborde et al., 2017). Lastly, among these indices, the $\Delta \,\bar{R\,R}$ index was significantly correlated with subjective/objective fatigue, physical load, and training level indicators, as ΔRMSSD and ΔLnRMSSD. Previous reviews were focused on interests and limits of HR and HRV measures on monitoring training status (Buchheit, 2014; Bellenger et al., 2016). If Buchheit considers resting HRV (more precisely RMSSD) as the HR measure more sensitive to fatigue (Buchheit, 2014), Bellenger et al. (2016) underlined some limits of this ANS status analysis. Indeed, in their meta-analysis, Bellenger et al. (2016) have observed that overload training had little effect on resting HRV due to various effects on vagally mediated HRV indices. The authors explained that the disagreement between studies may be the result of methodological issues. In addition to these methodological aspects, HRV analysis appears as a complex process due to different fatigue-induced alterations of HRV pattern (Schmitt et al., 2015). Given these limits of resting HRV, it is interesting to bring out new tools in ANS status evaluation. Our study therefore outlines a new indicator of stress/recovery status, the $\Delta \,\bar{R\,R}$. Based on our results, a low $\Delta \,\bar{R\,R}$, translating a low HR change between supine and standing positions, could mean a lack of post-exercise recovery. This observation is in accordance with the increase cardiac sympathetic modulation during supine rest and attenuated baroreflex response to change position observed by Uusitalo et al. (2000) in heavily trained females. The decrease observed here seems explained by an increase in supine HR without modification of standing HR (Barrero et al., 2019). This observation may be due to a decrease in parasympathetic and/or an increase in sympathetic HR influence (Schmitt et al., 2015). To our knowledge, the precise cause of this observation, decrease in sensitivity, and/or density of sinus cell membrane receptors or other one has not been formally demonstrated (Bellenger et al., 2016).

### Mathematical Model

As specified in the *Introduction*, we tested eight parameters that quantify physical load, training level, and fatigue indicators. The V̇O_2__*max*_ is widely used to assess both physical capacity and training level in endurance sports, but it is typically not repeatedly measured during a sporting season. Therefore, we included the training volume, summarized by the mean number of kilometers per week during the training period prior to the event. The chosen fatigue indicators reflect the internal load: RPE is commonly used to evaluate the perceived difficulty of an exercise and appeared strongly correlated to $\Delta \,\bar{R\,R}$, and the DOMS is specifically targeted at muscular fatigue; the perceived fatigue reflects general tiredness; quality of sleep and stress are associated with physical and mental fatigue.

We first studied the influence of the individual training level on the adaptation throughout the multistage cycling event. This training level depends on two main factors: the number of years of practice and the quality of training carried out during the weeks preceding the event. We noted no correlation between the number of years of practice and WB during the event. On the other hand (see Figure 2), except for the least trained cyclist, we observed a positive correlation between the training volume per week before the event and the adaptation all along the event. We therefore observed a predominant influence of recent training on the level of exercise tolerance during the multistage event.

Then, several multivariable models were tested. We showed that the indices $\Delta \,\bar{R\,R}$ and ΔLnRMSSD could be modeled linearly as a function of three main individual variables: training volume, V̇O_2__*max*_, and fatigue assessed through RPE and WB questionnaire. These indices appear to be relevant indicators of the adaptation ability along multistage events. However, we have noticed that linear models based on $\Delta \,\bar{R\,R}$ have more predictive power than those based on ΔLnRMSSD. Moreover, the individual $\Delta \,\bar{R\,R}$ and ΔLnRMSSD appear to be reliable indicators of both athlete’s training level and fatigue level. Indeed, we observed a positive correlation between $\Delta \,\bar{R\,R}$ (respectively, ΔLnRMSSD) and V̇O_2__*max*_, which reflects the training level and a negative correlation between $\Delta \,\bar{R\,R}$ (respectively, ΔLnRMSSD) and fatigue, RPE, WB, and maximal HR. The positive correlation we observed with the individual fitness and recovery of altered autonomic regulation after prolonged exercise confirms previous observations (Hautala et al., 2001; Fazackerley et al., 2019). Finally, the negative correlation with maximal HR confirms that the latter decreases with chronic endurance training (Bailey and Davies, 1999).

### Practical Applications

A decrease in the value of HRV indices is a marker of weak adaptability of the cardiovascular system to stress conditions that it faces (Michael et al., 2017); for example, the supine vagally mediated HRV parameters (RMSSD, total spectral power, and HF but not LF/HF) were lower in athletes identified in the fatigue state compared with the nonfatigue one (Plews et al., 2012; Schmitt et al., 2015). The active orthostatic test, a well-described marked physiological stress, is recommended to study HRV in athletes (Uusitalo et al., 2000; Hynynen et al., 2008; Schmitt et al., 2015).

From our results, it seems that the higher is $\Delta \,\bar{R\,R}$, HR difference between supine and standing positions, the better is the cardiovascular adaptability to orthostatic stress. Conversely, a low $\Delta \,\bar{R\,R}$ means stress-recovery imbalance as described in the paragraph *Respective Values of Heart Rate Variability Indices*. The negative impact of a Grand Tour on physical performance, mood, and WB of competitive cyclists is well reported, and a study performed with professional male cyclists during the Vuelta a España has noted that changes in supine HRV were inversely correlated to the exercise level (Earnest et al., 2004; Lastella et al., 2015; Rodríguez-Marroyo et al., 2017). Our results confirmed this observation. The impact of the preintervention physiological status on HRV alteration has also been proposed (Lastella et al., 2015; Rodríguez-Marroyo et al., 2017). Our data also underlined the importance of the pre-event training level to explain the HR adaptability change during a cyclist’s multistage event.

To our knowledge, it is the first time that the HR changes observed during an easy physiological test are modeled as a function of two physical parameters, training level and V̇O_2__*max*_, and one psychological parameter, fatigue. The model proposed allowed us to understand the ability to adapt to a repeated endurance exercise measuring the mean RR interval changes observed between supine and standing positions in well-trained female cyclists. If the measured $\Delta \,\bar{R\,R}$ is lower than predicted by the model, we could conclude that the imbalance of stress-recovery status is higher than perceived by the athlete in this context of a cyclist multistage event.

Thus, our findings support the use of $\Delta \,\bar{R\,R}$ monitoring to quantify training load, as $\Delta \,\bar{R\,R}$ can be directly predicted from fatigue and training level indicators. The use of $\Delta \,\bar{R\,R}$ monitoring can help coaches and athletes to make strategic decisions during a multistage long-duration event. It should be noted that the proposed model can be used with unsophisticated HR monitors (i.e., those recording only RR average values). This therefore makes it accessible to a majority of coaches and athletes, interested in the scientific approach of the training and performance monitoring. Finally, we also presented a model more accessible for coaches and team managers, connecting HR changes to a single indicator of fatigue. However, in accordance with its R^2^ adjusted and AIC values, the robustness of this model appears low, and it should be used with caution.

### Study Limitations

This study presents three main limitations. First, the small population sample studied can reduce the predictive power of the proposed model. However, the daily evolution of HRV in female athletes has been scarcely studied, and the model we proposed seems to be easy to use to follow the individual training and performance level. Second, our study focuses on well-trained female cyclists, and the validity of our model deserves to be confirmed in other sports. Specific studies are also needed in endurance male athletes, because HRV gender’s difference has been reported (Schäfer et al., 2015). Third, as some HRV indices are closely related and dependent on the individual’s breathing frequency during recording (No authors listed, 1996), it could have been relevant to impose controlled breathing (Gregoire et al., 1996), although this induces a mental activity and stress (Bernardi et al., 2000). At least, monitoring of respiratory rate could have been considered.

## Conclusion

From our data on well-trained female cyclists, we introduced a new indicator of post-endurance exercise recovery, the $\Delta \,\bar{R\,R}$ based on the change of mean HR observed during an orthostatic active test. This index is influenced by the training level and by the V̇O_2__*max*_ of the athlete. The proposed quantitative model can help to assess the adaptation ability of an athlete and thus to anticipate a decrease in endurance performance due to fatigue, particularly during a long-duration multistage cycling event. Investigating larger populations of athletes, included in other sports than cycling, represents an exciting perspective for future studies.

## Data Availability Statement

The original contributions presented in the study are included in the article/supplementary material, further inquiries can be directed to the corresponding author.

## Ethics Statement

The studies involving human participants were reviewed and approved by CHU Rennes, France. The patients/participants provided their written informed consent to participate in this study.

## Author Contributions

AB, SL, GC, FC, and FS performed the experimental conception and design. AB and SL performed the experiments. AB and ALC analyzed the data. AB, ALC, SL, FC, and FS written the manuscript. All authors read and approved the final manuscript.

## Conflict of Interest

The authors declare that the research was conducted in the absence of any commercial or financial relationships that could be construed as a potential conflict of interest.
